# Postpartum depression: How it differs from the “baby blues”

**DOI:** 10.1192/j.eurpsy.2021.1839

**Published:** 2021-08-13

**Authors:** M. Trigo

**Affiliations:** Psychiatry, Centro Hospitalar Universitário do Algarve, Faro, Portugal

**Keywords:** baby blues, breastfeeding, Postpartum depression, Antidepressants

## Abstract

**Introduction:**

Despite many signs and symptoms of depression get dismissed as normal physiologic changes associated with childbirth, depressive disorders are a common complication of pregnancy and postpartum period. The so-called “baby blues” have a minor functional impact and respond well to social support, whilst postpartum depression causes significant functional compromise, requiring more aggressive therapy. There is an extreme type of postpartum depressive disorder, postpartum psychosis, when patients present psychosis, mania, or thoughts of infanticide. It is imperative to promptly recognize and differentiate these entities, in order to minimize its impact on both mother and child. Antidepressant treatment may be necessary for some women, but risks and benefits should always be considered prior to institute pharmacotherapy.

**Objectives:**

To identify current approaches and evidence-based treatment options for postpartum depression.

**Methods:**

Review of the most recent literature regarding postpartum depression. The research was carried out through the Cochrane, UptoDate, PubMed, MedLine, LILACS and SciELO databases, using the terms “postpartum depression”, “baby blues” and “postpartum psychosis”, until December 2020.

**Results:**

Since both depression and antidepressant medications confer risk upon the infant, when postpartum depression develops, psychotherapy is usually the first-line treatment. Antidepressant treatment may be necessary, but its use during pregnancy and postpartum must be weighed carefully.

**Conclusions:**

In order to better prevent postpartum depression, recommendations include the use of screening instruments as a routine clinical practice during pregnancy and referral when necessary. Maternal depression has a severe impact on both mother and child, so mental health professionals have a very important role in reducing postnatal emotional complications.

**Disclosure:**

No significant relationships.

